# Lubricin in osteoarthritis: functions and therapeutic prospects

**DOI:** 10.3389/fimmu.2026.1790804

**Published:** 2026-05-15

**Authors:** Haochen Li, Guoliang Yi, Dan Zhou, Jiayi Zhou, Zhi Cui, Haowei Zhang, Zhiwei Chen

**Affiliations:** 1 Department of Orthopedic Surgery, The First Affiliated Hospital of University of South China, Hengyang, Hunan, China; 2 The Seventh Affiliated Hospital, University of South China (Hunan Provincial Veterans Administration Hospital), Changsha, Hunan, China

**Keywords:** chondrocyte metabolism, clinical transformation, joint lubrication, lubricin, osteoarthritis, synovial fibrosis, synovial immune microenvironment

## Abstract

Osteoarthritis (OA) represents a major global public health challenge, characterized by chronic pain and progressive loss of joint function. As the leading cause of disability among the elderly, it imposes a substantial socioeconomic burden worldwide. Pathologically, OA is increasingly understood as a systemic joint disorder that encompasses not only cartilage and subchondral bone, but also periarticular soft tissues and changes within the intra-articular microenvironment. Amid this complex, multi-tissue pathological milieu, lubricin(PRG4)—also known as superficial zone protein (SZP) or proteoglycan 4 (PRG4)—plays a critical chondroprotective role. Secreted predominantly by the superficial zone of articular cartilage and synovial fibroblasts, PRG4 contributes not only to boundary lubrication and the reduction of mechanical wear, but also to the maintenance of chondrocyte metabolic homeostasis, suppression of inflammatory signaling, modulation of immune cell polarization, and attenuation of synovial fibrosis. This review provides a comprehensive overview of the biological properties of PRG4 and elucidates its multifaceted protective mechanisms in joint homeostasis, including lubrication, metabolic regulation, immunomodulation, and antifibrotic activity—particularly through the CD44/TLR/NF-κB signaling pathway. Additionally, the therapeutic potential of PRG4 as a disease-modifying OA drug (DMOAD) is critically evaluated. Key translational challenges, such as issues related to interfacial adsorption stability and delivery system design, are also discussed. Collectively, this review aims to establish a theoretical framework and propose future research directions for developing novel biological therapies targeting OA.

## Introduction

Synovial joints are central to the locomotor function of the human skeletal system (1). Their biomechanical performance relies on the coordinated interaction of a complex system comprising articular cartilage, synovial fluid, the synovial membrane, and subchondral bone (2). Among these components, the highly efficient lubrication system formed by the cartilage surface and synovial fluid is essential for dissipating mechanical loads, minimizing inter-tissue friction and wear, and thereby preserving the long-term structural and functional integrity of the joint (3, 4). Osteoarthritis (OA), a prevalent degenerative joint disorder, is pathologically characterized by progressive degradation of articular cartilage, synovial inflammation, subchondral bone sclerosis, and impaired joint lubrication (5, 6). Global epidemiological surveys indicate a persistent rise in OA incidence (7). By 2020, OA had affected 595 million people, accounting for 7.6% of the worldwide population, and imposed a heavy medical and socioeconomic burden (8). However, effective interventions capable of delaying or reversing disease progression remain elusive (9), underscoring the need to deepen our understanding of joint lubrication and protective mechanisms.

Lubricin, encoded by the *PRG4* gene, is a critical macromolecule abundantly present in synovial fluid and on the surface of articular cartilage (10).Early studies have shown that PRG4 and surface zone protein (SZP) are homologous products of the megakaryocyte-stimulating factor (MSF) gene and share a common molecular origin. Therefore, they do not represent distinct proteins, but rather result from differential gene expression and post-transcriptional processing within the same precursor system (11, 12).Initially recognized for its boundary lubricating role, PRG4’s characteristic bottle-brush structure underlies its biomechanical function (13, 14). This architecture enables PRG4 to specifically anchor to cartilage surfaces and form a hydrated molecular film, thereby drastically reducing friction coefficients and ensuring near-frictionless joint motion, especially under high mechanical load (15, 16). Beyond its mechanical role, accumulating evidence has revealed that PRG4 also functions as a multifunctional signaling molecule central to maintaining the homeostasis of the intra-articular microenvironment (17). PRG4 has been reported to play a key role in maintaining cartilage homeostasis and protecting cartilage surfaces from friction-induced damage (18, 19). In addition, accumulating evidence suggests that PRG4 exerts anti-inflammatory effects through suppression of pro-inflammatory signaling pathways (20). PRG4 has also been implicated in the modulation of immune responses, including the regulation of macrophage activation and innate immune signaling pathways (21). Furthermore, PRG4 may contribute to the regulation of synovial fibroblast activation and fibrotic responses (22). These discoveries have redefined PRG4 not merely as a “lubricating molecule,” but rather as a “multifunctional guardian” of joint integrity.

The indispensable role of PRG4 is further underscored by genetic evidence: mutations in *PRG4* result in camptodactyly-arthropathy-coxa vara-pericarditis (CACP) syndrome (23), a rare autosomal recessive disorder characterized by congenital joint contractures, non-inflammatory arthropathy with pronounced synovial hyperplasia, and early-onset joint degeneration (24). This condition provides compelling confirmation of the pathological consequences of PRG4 deficiency. Disorders such as OA, although *PRG4* transcription may be upregulated as a compensatory response (25), the functional PRG4 protein in synovial fluid and at cartilage interfaces is often markedly reduced or structurally altered (26). This disconnect between gene expression and protein function is increasingly recognized as a key contributor to OA progression.

Given the growing interest in lubrication biology, several recent reviews have summarized its role in OA, particularly highlighting its biological functions, upstream regulatory mechanisms, and therapeutic potential (27). However, the role of lubricants in coordinating immune inflammatory processes in the joint microenvironment has not yet been systematically understood. In this review, we focus more on the immunomodulatory and anti-inflammatory properties of lubricants, and further integrate these functions with their roles in joint biomechanics, chondrocyte metabolism, and synovial fibrosis. Collectively, we propose that lubricants represent a critical nexus linking mechanical stress, inflammation, and tissue remodeling in OA pathogenesis.

## Biological characteristics of PRG4

**Figure 1:**
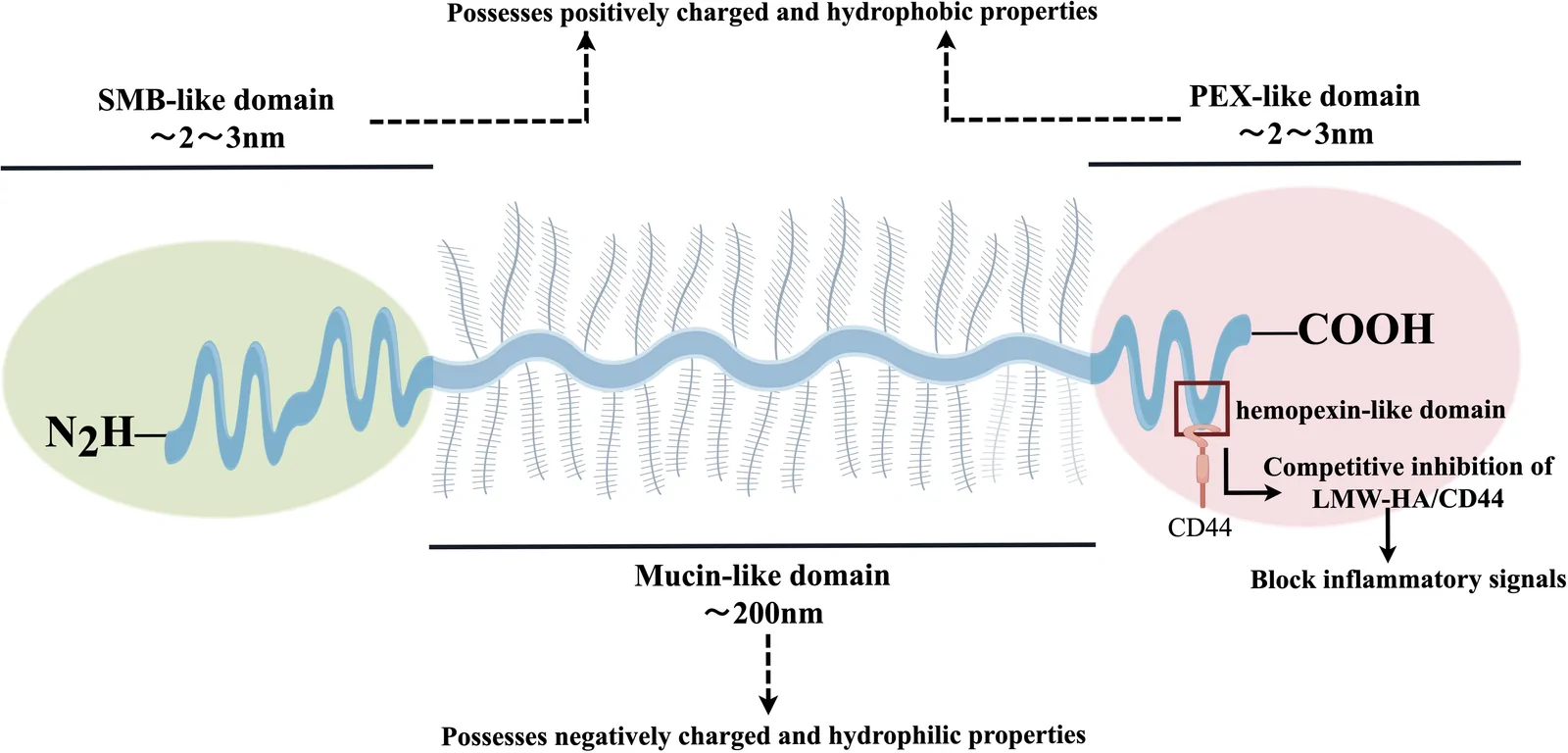
Schematic diagram of the lubricin molecular structure. Schematic diagram of the lubricin molecular structure. lubricin is a mucin-like glycoprotein characterized by a “bottle-brush” architecture. This structure comprises a central, heavily O-glycosylated mucin domain that is hydrophilic and negatively charged, responsible for boundary lubrication and anti-adhesion. This domain is flanked by two globular domains: an N-terminal somatomedin B-like (SMB-like) domain and a C-terminal hemopexin-like (PEX-like) domain. These termini are positively charged and hydrophobic, mediating surface anchoring and cell communication. The C-terminal domain also competes with low-molecular-weight hyaluronic acid (LMW-HA) for CD44 binding, thereby inhibiting inflammatory signals.

PRG4 is a mucin-like glycoprotein encoded by the PRG4 gene (28), which is also recognized as superficial zone protein (SZP) due to its predominant synthesis and secretion by superficial zone chondrocytes and synovial fibroblasts (29). The molecular structure of PRG4 comprises a glycosylated polypeptide chain approximately 200 ± 30 nm in length, featuring non-glycosylated regions at both ends and a centrally located, highly glycosylated functional domain (30, 31) (**Figure 1**). The modular PRG4/SZP/MSF protein consists of multiple exons that give rise to distinct domains. The highly glycosylated central domain of PRG4, encoded by exons 6–9, is primarily responsible for boundary lubrication; the N-terminal and C-terminal domains, meanwhile, share structural homology with extracellular matrix-binding domains, which facilitate cell-matrix interactions and protein anchoring. Notably, the PRG4 gene undergoes alternative splicing involving exons 2, 4, and 5, generating multiple distinct PRG4 isoforms with varied structural and functional properties (11, 12). This molecular heterogeneity indicates that PRG4 does not act as a single uniform molecule but rather functions as a family of glycoproteins with context−dependent activities tailored to diverse tissue microenvironments. The special structural of PRG4 allows its widespread distribution in synovial fluid and on cartilage surfaces, where it plays a central role in maintaining joint lubrication and tissue homeostasis (32). Beyond the joint system, PRG4 is widely present in tissues that require mechanical protection, such as tendons, ligaments, and intervertebral discs (33, 34). Additionally, varying degrees of expression have been detected in organs including the liver, heart, and lungs (17, 35).

The expression and activity of PRG4 are under precise multi−level regulatory control. At the transcriptional level, PRG4 transcription is governed by key transcription factors including Foxo1 and Creb5, which integrate upstream signals from the TGF−β/Wnt signaling axis to fine−tune expression levels (27). At the post-translational modification level, the degree of glycosylation directly influences its protein stability and functional activity (26, 36). Mechanical loading exerts a biphasic regulatory effect on PRG4 synthesis: moderate physiological mechanical stimulation promotes PRG4 expression and secretion, whereas excessive or abnormal mechanical stress leads to transcriptional repression and functional impairment (37, 38). It is worth noting that PRG4 can regulate the expression of various factors, and these factors, in turn, can influence the expression levels of PRG4. Pro-inflammatory cytokines such as IL-1 inhibit the synthesis of PRG4, while growth factors such as TGF-β and IGF-1 promote its expression (11, 39). Disruption of PRG4’s intricate regulatory mechanisms is associated with the development of various pathological conditions across different tissues, including joint degeneration and ocular surface disorders.

Pathologically, loss−of−function mutations in the PRG4 gene cause camptodactyly−arthropathy−coxa vara−pericarditis (CACP) syndrome, a rare autosomal recessive hereditary disorder (40). Typical clinical features of CACP include congenital or early-onset flexion contractures, non-inflammatory arthropathy with synovial hyperplasia, and progressive joint degeneration (41). Joint structure is typically intact at birth but develops progressive synovial fibrosis and cartilage degeneration with age (42). Some patients also manifest progressive coxa vara deformity and non-inflammatory pericardial or pleural effusions (24, 43). Beyond the hereditary CACP syndrome, abnormal PRG4 expression is closely associated with acquired joint diseases such as OA (44). During OA progression, dynamic changes in PRG4 exhibit cross-tissue regulatory imbalance: PRG4 expression is significantly upregulated in the synovium (25), while it shows a downregulation trend in synovial fluid and cartilage (45). This heterogeneous alteration arises from multiple pathological factors including synovial proliferation, elevated pro−inflammatory cytokines, abnormal mechanical loading distribution, and progressive cartilage wear, collectively disrupting joint lubrication and homeostatic balance.

## Biological functions of PRG4 in osteoarthritis

### Boundary lubrication and anti−adhesion

As a key functional molecule in synovial fluid, PRG4 achieves boundary lubrication and anti-adhesion functions through its unique three-domain structure (46, 47). Specifically, its centrally located, highly glycosylated domain interacts synergistically with the globular domains at both ends to collectively form a stable functional barrier at biological interfaces (26, 48). The central lubricating domain contains a 76−amino−acid tandem repeat sequence enriched in O−glycosylation sites, with dominant sugar components including N−acetylgalactosamine (GalNAc), galactose (Gal), and sialic acid (NeuAc) (29, 31). The high density of negatively charged and highly hydrated sugar chains generates strong steric repulsion and osmotic effects, forming a compact hydration layer that markedly reduces the friction coefficient between opposing cartilage surfaces during joint motion (14, 19),and exhibits outstanding anti-adhesive properties (49). Meanwhile, the high molecular weight of this central domain facilitates tight and persistent adsorption to the cartilage surface, ensuring a sustained lubrication function (16, 29).

In contrast to the central glycosylated region, PRG4’s two ends comprise globular protein-like subdomains: an N-terminal SMB-like domain and a C-terminal PEX-like domain (18), with structural similarity to vitronectin (28). These domains mediate specific interactions with cartilage ECM components and cell surface receptors, enabling precise localization and stable anchorage of PRG4 molecules (29) (50).The overall molecular structure of PRG4 exhibits a classic “bottle brush” polymer configuration, featuring a centrally located, highly glycosylated mucin-like domain that resembles “bristles,” flanked by globular domains at both ends that resemble “caps” (15). This “bristle-cap” combination achieves perfect functional division and synergy: the central hydration layer reduces friction, while the terminal anchoring domains ensure stable molecular attachment. During joint movement, this structure forms an efficient “liquid cushion layer” between the contacting surfaces, significantly reducing direct contact and achieving lubrication (29). Furthermore, the interfacial effectively prevents abnormal protein deposition and excessive synovial cell adhesion, thereby protecting cartilage integrity and maintaining normal joint function through multiple mechanisms (51).

### Immune inflammation suppression

In addition to its well-established role in boundary lubrication and surface protection, PRG4 also exerts important anti-inflammatory and immunomodulatory effects within the joint. Increasing evidence suggests that PRG4 is not merely a passive biomechanical molecule, but an active regulator of the synovial immune microenvironment. The binding of lubricants to CD44 is an important way for them to inhibit synovial inflammatory signals, CD44 is a widely expressed cell surface receptor that plays a central role in joint inflammation and the degradation of the extracellular matrix (52, 53). The biological effects of its ligand, hyaluronic acid (HA), exhibit distinct molecular weight dependence: High molecular weight HA (HMW-HA) binds to CD44 to exert anti-inflammatory and chondroprotective effects (54). Conversely, under pathological conditions, the degradation of HMW-HA generates lower molecular weight HA (LMW-HA), which activates the CD44/NF-κB signaling pathway, promotes the release of inflammatory mediators, and exacerbates cartilage destruction (55, 56). Studies indicate that PRG4 binds to CD44 with high affinity, competitively inhibiting the interaction between LMW-HA and CD44, thereby blocking pro-inflammatory signaling (57). This action effectively suppresses NF-κB pathway activation in a concentration-dependent manner, significantly reducing joint inflammatory responses. Notably, when PRG4 removes glycation modifications, it exhibits enhanced CD44 binding capacity, suggesting potential regulatory roles of glycan structures in protein function (58). Importantly, because CD44 signaling participates not only in synoviocyte activation but also in leukocyte adhesion, trafficking, and inflammatory amplification within the synovium, PRG4–CD44 interaction may have broader consequences for the organization of the local immune niche than simple suppression of a single inflammatory axis (58, 59).

Beyond CD44, PRG4 directly targets Toll-like receptors (TLRs), particularly TLR2 and TLR4, thereby inhibiting their activation. TLRs serve as key receptors that recognize pathogen-associated molecular patterns (PAMPs) and damage-associated molecular patterns (DAMPs), playing a central role in initiating innate immune responses (60, 61). Experiments have shown that PRG4 binds to TLR2/4 in a concentration-dependent manner, thereby inhibiting the recruitment of downstream adaptor proteins such as MyD88. This suppression of TLR2/4 signaling blocks IKK complex activation, prevents IκBα degradation, and reduces NF-κB nuclear translocation, ultimately suppressing the transcription of inflammatory genes. In animal models, PRG4 effectively inhibits macrophage polarization toward the M1 phenotype through this mechanism, reducing levels of proinflammatory factors such as IL-1β and IL-6, thereby alleviating synovial inflammation and cartilage structural damage (22, 27). Given the central role of TLR-driven macrophage activation in shaping the inflammatory tone of OA synovium, this receptor-level antagonism suggests that PRG4 can influence not only soluble inflammatory mediators but also the cellular composition and functional state of the synovial immune microenvironment (22, 59).

As central integrators of inflammatory signaling, synovial macrophages coordinate processes such as debris clearance, immune surveillance, and the resolution of inflammation (62, 63).Previous studies have shown that a lack of PRG4 can disrupt the homeostasis of synovial macrophages, leading to the abnormal activation and accumulation of pro-inflammatory M1-like macrophages; conversely, exogenous supplementation with PRG4 can inhibit this process and promote their conversion to an anti-inflammatory phenotype (64).Based on this, the function of PRG4 has gradually expanded from that of a traditional “lubricating molecule” to that of a key ECM signaling molecule involved in synovial immune regulation; it can even be regarded as a key regulator of synovial macrophage homeostasis. Recent studies have further revealed that PRG4 not only regulates the polarization of macrophages but also plays a significant role in modulating their metabolic processes. A 2024 study demonstrated that in the absence of PRG4 function, synovial macrophages are more prone to adopt a pro-inflammatory state, accompanied by an enhanced xanthine oxidase (XO)–HIF-1α axis, increased glycolysis, and elevated reactive oxygen species (ROS) production (65, 66).These metabolic changes not only reflect the inflammatory state itself but may also form a positive feedback loop that sustains the M1-like phenotype by stabilizing HIF-1α signaling and promoting NF-κB-mediated transcriptional programs, thereby amplifying the local inflammatory response (67–69). In contrast, recombinant human PRG4 (rhPRG4) can reverse this pro-inflammatory metabolic reprogramming, suggesting that it plays a crucial role in the regulation of immune metabolism (65).In addition, PRG4 plays a crucial protective role in maintaining the Cx3CR1^+^/TREM2^+^ synovial barrier-like macrophage population (65).This cell population is believed to act as a barrier, maintaining the structural integrity of the synovial lining and limiting the entry of peripheral immune cells into the joint cavity (70).Therefore, the protective effect of PRG4 on this subpopulation is not limited to cell survival but also involves the overall regulation of the synovial structural barrier and its immune-isolating function. In addition to its role in innate immune regulation, PRG4 is also involved in adaptive immune regulation. Evidence suggests that PRG4 may act as a candidate autoantigen in joint diseases such as rheumatoid arthritis. In this context, disease progression may result not only from excessive immune activation but also from impaired immune tolerance. Specifically, a lack of response by regulatory T cells (Tregs, CD4^+^ CD25^+^) to PRG4-associated epitopes may disrupt the immune homeostasis of synovial tissues. This hypothesis is supported by the fact that tissues commonly affected in rheumatoid arthritis—such as synovial joints, bursae, tendon sheaths, and the pericardium—are all rich in PRG4, and its expression is influenced by mechanical friction (71).

Collectively, PRG4 regulates joint inflammation through a multi−level synergistic network: at the receptor level, it blocks inflammatory signaling initiation by competitively binding CD44 and antagonizing TLR2/4 activation; at the transcriptional level, it suppresses NF−κB−driven pro−inflammatory gene expression; at the cellular level, it normalizes macrophage polarization and metabolic reprogramming while sustaining protective resident macrophage subsets. Through this multi−layered regulation, PRG4 redirects the synovial microenvironment from a vicious “inflammation–destruction–fibrosis” cycle toward a homeostatic “homeostasis–barrier–repair” state, and may further influence the dynamic balance between innate and adaptive immunity.

### Mechano-inflammatory coupling in lubricin deficiency

In OA pathogenesis, abnormal mechanical loading and inflammation are not independent events, but are closely intertwined in a self-amplifying “mechanical-inflammatory cycle” (72, 73). PRG4 serves as a central protective role; their absence should not be simply interpreted as a loss of lubrication or mere activation of inflammation, but rather as a pathological state that promotes the transition from biomechanical dysfunction to chronic aseptic inflammation.

At the mechanical level, PRG4 typically reduce friction on the cartilage surface and help buffer excessive shear and stress transmitted to superficial chondrocytes (19). When PRG4 is reduced or impaired, friction on the cartilage surface increases, leading to abnormal stress concentrations and enhanced mechanical stimulation of chondrocytes (74). These pathological mechanical signals activate mechanotransduction pathways, including mechanosensitive ion channel signaling, promoting Ca²^+^ influx and triggering downstream inflammatory and catabolic responses (75, 76). In this way, the loss of PRG4 lowers the threshold for converting abnormal joint mechanics into pro-inflammatory intracellular signals (77, 78). Mechanically induced cellular stress further exacerbates damage to the cartilage surface and degradation of the extracellular matrix (78, 79). As tissue damage progresses, the damaged cartilage releases endogenous danger-associated molecular patterns (DAMPs), which serve as key molecular mediators linking mechanical injury to innate immune activation (45, 80). These DAMPs activate Toll-like receptors (TLRs) on synovial cells and macrophages, particularly TLR2 and TLR4, thereby driving downstream inflammatory signaling and further increasing the production of pro-inflammatory mediators (81). Consequently, the initial biomechanical abnormalities evolve into a more widespread inflammatory response, which in turn accelerates additional cartilage damage, reinforcing the pathological cycle. PRG4 can bind directly to TLR2 and TLR4, thereby antagonizing DAMP-mediated receptor activation and limiting downstream inflammatory amplification (22, 44, 82). Thus, PRG4 exerts a dual decoupling effect on the mechanical-inflammatory coupling: it blocks the initiation of inflammation at the mechanical level and suppresses its amplification at the immune level. This dual action positions PRG4 as a key molecular barrier against the vicious cycle of friction-induced damage, innate immune activation, and progressive joint degeneration in OA.

### Metabolic regulation of chondrocytes

Degeneration of articular cartilage is a hallmark pathological feature of OA (83). Beyond external abnormal mechanical loading, metabolic dysregulation within chondrocytes themselves—characterized by weakened anabolic processes and heightened catabolic activity—constitutes a core mechanism driving matrix degradation and disease progression (84, 85). Specifically, the reduced expression of anabolic markers (e.g., Acan, Tgfb1) in chondrocytes, coupled with the upregulation of catabolic enzymes (e.g., Col10a1, Mmp13), leads to the disruption of the type II collagen network and the loss of proteoglycans, ultimately accelerating OA progression (86, 87).

As a key secretory factor of superficial chondrocytes and synovial fibroblasts, PRG4 maintains chondrocyte metabolic balance through bidirectional regulation: enhancing anabolism and restraining catabolism. Transgenic mouse studies have shown that PRG4 overexpression significantly suppresses catabolic gene expression and chondrocyte hypertrophy, exerting robust protective effects in age−related and post−traumatic OA models. PRG4 also attenuates hypoxia−induced cartilage degeneration by positively regulating Hif3α, a negative modulator of HIF−1α/2α (40). Subsequent work further confirmed the anabolic role of PRG4: intra−articular delivery of PRG4−expressing helper−dependent adenovirus effectively promotes chondrogenic gene expression, including Acan and TGF−β1, and mitigates post−traumatic cartilage damage (87).

Furthermore, PRG4’s bidirectional regulation of cartilage metabolism is closely linked to its function in maintaining joint mechanical homeostasis (88). Under physiological conditions, appropriate mechanical stimulation promotes anabolic processes in articular cartilage (89). However, in OA, abnormal mechanical loads translate into pathological signals (90, 91). On one hand, they activate downstream inflammatory pathways by stimulating sensors such as integrins and mechanosensitive ion channels (92). On the other hand, they directly induce oxidative stress responses, collectively leading to the upregulation of catabolic enzymes, including MMP13 and ADAMTS5 (93–95).

As a key intra-articular lubricant, PRG4 reduces joint friction coefficients by forming boundary lubrication films on articular surfaces (14). Lubricant indirectly modulates the anabolic/catabolic balance in chondrocytes by attenuating pathological signal transmission (96). This indirect mechanical regulation synergizes with PRG4’s modulation of gene expression related to cartilage anabolism and catabolism, forming a multi-tiered protective network that helps preserve cartilage homeostasis.

### Inhibition of synovial fibrosis

Following its role in maintaining cartilage metabolic homeostasis, PRG4 also participates in regulating other key pathological processes in OA—synovial fibrosis. In the complex pathophysiology of OA, synovial fibrosis is a key mechanism leading to joint dysfunction and increased pain (97), characterized by the excessive deposition of extracellular matrix (ECM) in synovial tissue, loss of tissue elasticity, and progressive joint stiffness (98, 99). Synovial fibrosis in OA is closely associated with persistent low-grade inflammation, which acts as an important upstream driver of fibroblast activation and ECM deposition (45, 100).

At the cellular level, PRG4 directly acts on synovial fibroblasts (SFs), synergistically inhibiting their pathological activation and function via multiple pathways, thereby intervening in the initiation and progression of fibrosis. Specifically, PRG4 significantly suppresses fibroblast-myofibroblast transformation (FMT) induced by factors such as TGF-β (101, 102). Studies confirm that exogenous PRG4 treatment in synovial cells derived from OA patients effectively reduces α-smooth muscle actin (α-SMA) expression and stress fiber formation, blocking the shift toward a highly contractile, secretory phenotype (101). In addition, PRG4 significantly suppresses the abnormal proliferation of SFs. *In vitro* experiments demonstrate that PRG4—whether administered as recombinant human PRG4 (rhPRG4) to TGF-β-stimulated cells or in inflammatory co-culture models with macrophages—markedly reduces cellular proliferation activity, thereby mitigating fibrosis exacerbated by increased cell numbers (103). PRG4 also imposes restrictions on SF migration capacity. Evidence from scratch assays and other studies consistently demonstrates that PRG4 treatment markedly inhibits cell migration (101), indicating its efficacy in preventing the dissemination and aggregation of fibrotic cells within synovial tissue. These findings suggest that by which PRG4 directly targets synovial fibroblasts to suppress fibrosis through three interrelated pathways: inhibiting transformation, proliferation, and migration.

In addition to its direct effects on synovial fibroblasts, PRG4 may also indirectly attenuate fibrotic progression by modulating the inflammatory microenvironment. As mentioned above, PRG4 suppresses pro-inflammatory signaling and promotes macrophage polarization toward an anti-inflammatory phenotype, thereby reducing the levels of key profibrotic cytokines such as IL-1β, TNF-α, and TGF-β. Since these mediators are closely involved in fibroblast activation, fibroblast-to-myofibroblast transition, and ECM overproduction, their reduction provides a plausible mechanistic link between inflammation resolution and fibrosis inhibition (104, 105). From a molecular mechanism perspective, PRG4’s inhibition of fibrosis ultimately manifests as the regulated downregulation of key fibrosis-related protein expression, a process dependent on the regulation of its receptor-mediated signaling pathway. Studies indicate that PRG4 specifically binds to receptors such as CD44 and Toll-like receptors (TLR2/TLR4) on synovial cell surfaces, thereby inhibiting the nuclear translocation and transcriptional activity of downstream NF-κB signaling (58, 82). Because NF-κB plays a central role in both inflammatory responses and fibrosis-related gene expression, its inhibition by PRG4 may help disrupt the pathological interaction between inflammation and fibrosis (106, 107). Moreover, PRG4 also helps preserve ECM homeostasis. By regulating the synthesis and composition of key matrix components produced by synovial fibroblasts, including type I collagen and glycosaminoglycans, PRG4 helps maintain normal synovial structure and limits fibrosis associated with excessive ECM deposition or matrix imbalance (100, 108, 109).Animal models further support this mechanism: in PRG4-deficient mice, the expression of fibrosis markers such as α-SMA, type I collagen, and PLOD2 is significantly increased, whereas reintroduction of PRG4 reverses these pathological changes (101).

**Figure 2:**
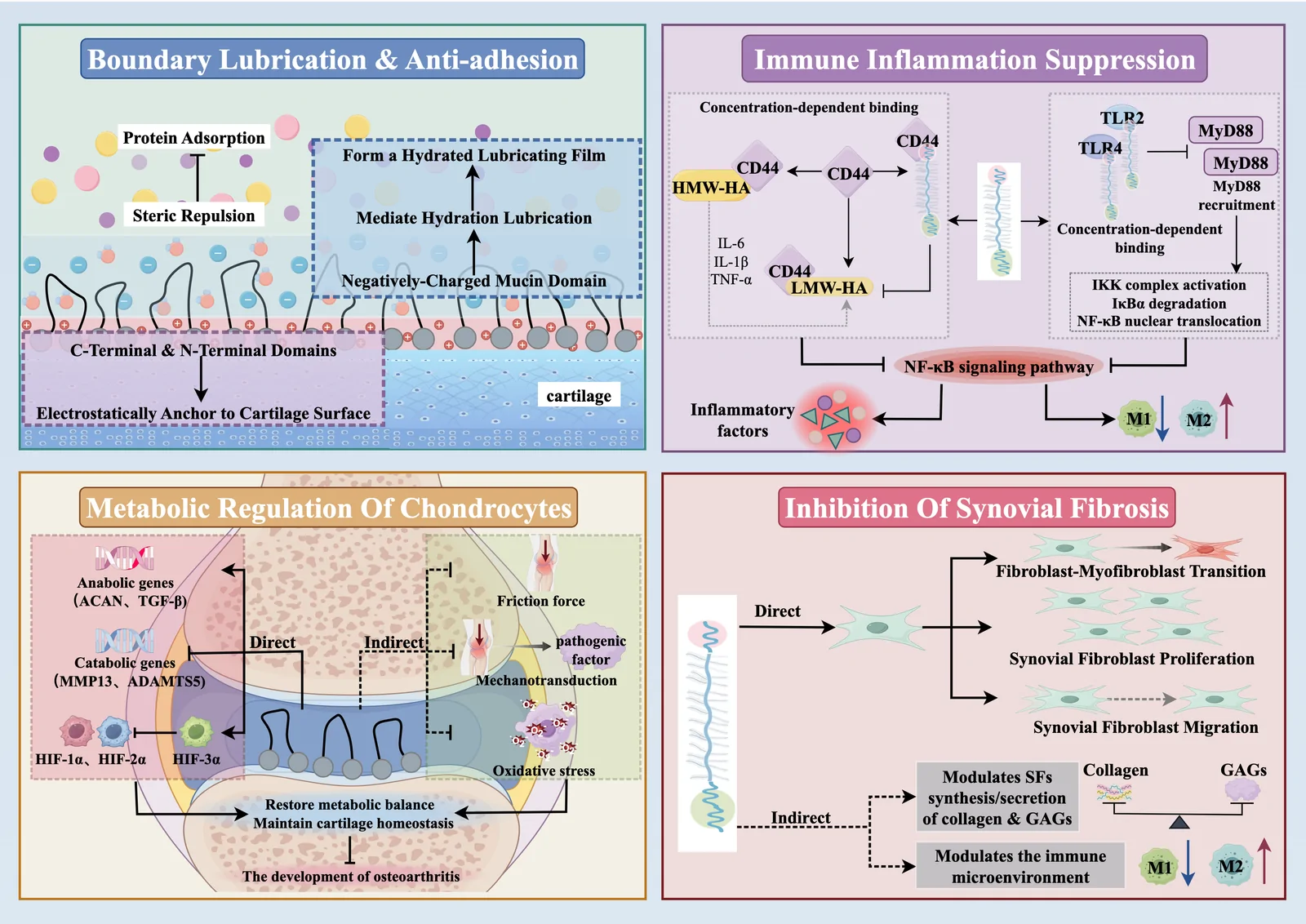
Schematic overview of the multifaceted protective mechanisms of lubricin in joint.lubricin exerts integrated protective effects in osteoarthritis through four major functional axes (1). Boundary lubrication and anti−adhesion: lubricin adsorbs onto the cartilage surface via its terminal domains and forms a hydrated, negatively charged mucin-like brush layer, generating steric repulsion and hydration lubrication to reduce friction, prevent protein deposition, and inhibit cell adhesion (2). Immune inflammation suppression: lubricin binds to CD44 and competitively inhibits the pro-inflammatory effects of low-molecular-weight hyaluronic acid (LMW-HA), while also directly antagonizing TLR2/4 signaling, thereby blocking MyD88 recruitment and suppressing NF-κB activation. This results in reduced production of inflammatory cytokines and promotes macrophage polarization toward the anti-inflammatory M2 phenotype (3). Metabolic regulation of chondrocytes: lubricin directly and indirectly maintains chondrocyte metabolic homeostasis by enhancing anabolic gene expression (e.g., ACAN, TGF-β) and inhibiting catabolic factors (e.g., MMP13, ADAMTS5), while attenuating Mechan transduction and oxidative stress induced by excessive friction, ultimately preserving cartilage integrity (4). Inhibition of synovial fibrosis: lubricin suppresses fibroblast-to-myofibroblast transition, proliferation, and migration of synovial fibroblasts, and modulates extracellular matrix synthesis (collagen and glycosaminoglycans). Additionally, by reshaping the immune microenvironment and inhibiting NF-κB signaling, it indirectly attenuates fibrosis progression. Collectively, lubricin functions as a central regulator linking biomechanical lubrication, immune modulation, metabolic balance, and antifibrotic processes, thereby maintaining joint homeostasis and slowing osteoarthritis progression.

Overall, these findings demonstrate that PRG4 acts as a multifunctional regulator of joint homeostasis, integrating mechanical protection, metabolic regulation, immune modulation, and antifibrotic effects within the joint microenvironment(**Table 1**; **Figure 2**).

**Table 1:** Summary of the multifaceted biological functions of lubricin.

Functional category	Key mechanisms	Biological effects	Related signaling pathways/molecules	References
Boundary lubrication & anti-adhesion	Forms a macromolecular lubrication film on the cartilage surface via a “molecular brush” structure	Reduces the coefficient of friction and prevents cartilage wear	Central tunnel domain (lubricin), N/C-terminal globular domains (heparin-binding)	Yunsup Lee et al.2018
Chondrocyte metabolism regulation	Upregulates anabolic markers (e.g.Acan,TGF-β1); Downregulates catabolic markers (e.g.Mmp13, Runx2)	Delays cartilage degradation and inhibits chondrocyte hypertrophy	HIF-2α, TGF-β/Wnt signaling pathways	Ruan MZ et al.2013 Adrianne Stone et al.2019
Immunomodulation & anti-inflammation	Competitively binds CD44, blocking the pro-inflammatory effects of LMW-HA	Inhibits NF-κB activation and alleviates joint inflammation	CD44, NF-κB	Afnan Al – Shari et al.2015 Ali Alquraini et al.2015 Qadri M et al.2021
	Directly antagonizes TLR2/4 activation	Prevents MyD88 degradation, suppresses NF-κB activity, and reduces pro-inflammatory cytokines	TLR2/4 MyD88 NF-κB	
	Modulates macrophage polarization and infiltration	Suppresses M1 polarization, promotes M2 polarization, and reduces inflammatory cytokines (e.g.IL-1β,IL-6)	NF-κB CCL2 CXCL10	
Anti-fibrosis	Direct: Inhibits fibroblast-to-myofibroblast transition (reducing α-SMA expression), proliferation, and migration	Prevents the initiation of fibrosis	CD44/TLR/NF-κB pathway	Marwa Qadri et al.2020 Qadri M et al.2021

## Clinical translation prospects and challenges of PRG4

Despite the well−established homeostatic and chondroprotective functions of PRG4, clinical translation of PRG4−based therapies for OA remains challenging. Preclinical studies consistently demonstrate that recombinant PRG4 supplementation and PRG4 gene therapy enhance joint lubrication, reduce synovial inflammation, mitigate cartilage degradation, and improve joint function in experimental OA models. However, these promising preclinical findings have not yet translated into standardized, widely available clinical interventions (26, 37, 51).

One of the central obstacles in translating PRG4-based strategies lies in its retention at the joint interface, since its boundary-lubricating capacity depends on effective adsorption to cartilage or other articulating surfaces. Experimental evidence indicates that this adsorption process, along with subsequent brush-layer formation and overall lubrication performance, is strongly influenced by surface chemistry and the underlying properties of the substrate (51, 110), Because PRG4 does not act in isolation but interacts with components of the cartilage matrix (including type II collagen), this suggests that damage to the tissue surface (as seen in pathological conditions) may directly impair its ability to anchor itself and function effectively (37). Furthermore, in cases of OA, the proteolytic cleavage of PRG4 reduces its lubricating properties, thereby exacerbating the problem and potentially diminishing the persistence of functional lubricants on the joint surface (26). Taken together, these findings offer a more nuanced explanation for the limited efficacy of simple intra-articular supplementation: the challenge lies not only in the dosage but also in whether the lubricant can maintain a stable and functionally organized presence within the pathologically altered interface. Another factor hindering the translation of research findings into clinical practice is the limited predictive capability of current detection systems. Taking PRG4 as an example, its friction and lubrication properties vary depending on the contact surfaces used in experiments, and measurable differences have been observed between cartilage-cartilage and cartilage-glass models (111), this highlights the significant shortcomings of simplified *in vitro* tribological systems in reflecting the mechanical and interfacial complexity of human joints. Under these circumstances, the therapeutic effects observed in reductionist models may lead to overly optimistic expectations regarding clinical outcomes. Furthermore, the inherent structural complexity of the lubricant itself—as a large, highly glycosylated mucin-like glycoprotein—means that PRG4 belongs to a class of molecules that are difficult to produce via recombinant methods and for which the quality of the glycosylation profile is hard to precisely control. This directly impacts product consistency, scalability, and formulation development (112).

Efforts to address the short intra-articular residence time of PRG4 have led to the development of viral gene-delivery strategies designed to sustain PRG4 expression within the joint space. In mouse models of post-traumatic OA, approaches based on helper-dependent adenoviral or AAV-mediated gene transfer have demonstrated chondroprotective effects while maintaining prolonged expression profiles (40, 87), suggesting a promising direction at least at the preclinical level. Yet these advantages invite a more cautious evaluation, as the same features that enable sustained endogenous production are accompanied by challenges inherent to gene therapy platforms—vector immunogenicity, difficulties in dose regulation, regulatory hurdles, and lingering uncertainties about long-term expression dynamics across the diverse cellular landscape of joint tissues (87). For PRG4, the situation becomes even more intricate, given that its functional efficacy depends not simply on how much is produced, but on whether it undergoes appropriate post-translational modifications (112) and achieves correct spatial localization (51). In light of this, while viral vectors offer a highly attractive approach to achieving persistence, they have not fully addressed the deeper issue: ensuring that the lubricant not only remains present but is also properly organized into a bioactive layer capable of functioning effectively within the complex environment of diseased human joints. In contrast to gene-based strategies, biomaterial coatings and surface-engineering approaches engage with a different aspect of the translational challenge by acting directly at the interface, where spatial control becomes critical. Insights from studies on PRG4 self-assembly (51), along with the development of PRG4-inspired or PRG4-recruiting surfaces (32), suggest that thoughtful material design can improve lubrication and limit fouling, particularly at cartilage–biomaterial or cartilage–metal boundaries (32). This perspective is reinforced by observations that PRG4 reduces friction more effectively than other key synovial fluid components in both cartilage–cartilage and cartilage–metal articulations, highlighting its particular relevance in the context of implant-associated applications (113). Still, the promise of these strategies is tempered by practical limitations: coating stability under cyclic mechanical stress, vulnerability within the protease-rich environment of osteoarthritic joints, and incomplete conformity to the irregular topography of native cartilage all pose challenges to sustained performance (32, 51). When comparing the two conversion strategies, biomaterial coatings offer precise interface targeting but struggle to achieve long-term biocompatibility (32), whereas viral vectors achieve more durable expression but lack spatial specificity and introduce greater regulatory complexity. It is precisely this tension between persistence and control that defines the current bottleneck in translating PRG4-based therapies into reliable clinical solutions.

Taken together, the translational barrier of PRG4−based therapy lies in balancing four key factors: intra−articular retention, spatial presentation, molecular functional integrity, and long−term durability. Future progress will likely come from combinatorial strategies that integrate biomaterial−enhanced anchoring, targeted delivery systems, and protein engineering to improve stability and half−life, alongside more physiologically relevant joint models for reliable therapeutic evaluation.

## Summary and outlook

PRG4 is a core multifunctional molecule that maintains articular joint homeostasis by integrating boundary lubrication, immune modulation, chondrocyte metabolic regulation, mechano−inflammatory decoupling, and anti−fibrotic activity. Its unique “bottle−brush” molecular structure underpins its dual role as both a biomechanical lubricator and a signaling regulator: the central highly glycosylated mucin−like domain forms a hydration layer to reduce friction and prevent adhesion, while the N− and C−terminal globular domains mediate cartilage anchorage and receptor−dependent cellular crosstalk.

Accumulating evidence has expanded the role of PRG4 from a passive lubricating molecule to an active regulator of the joint microenvironment. It preserves chondrocyte metabolic homeostasis by promoting anabolic gene expression and suppressing catabolic and hypertrophic programs; it suppresses synovial inflammation by blocking CD44− and TLR2/4−mediated NF−κB activation and normalizing macrophage polarization and metabolism; it uncouples pathological mechano−inflammatory cycles; and it inhibits synovial fibrosis by directly restraining synovial fibroblast activation and indirectly reducing pro−fibrotic inflammatory signals. In hereditary CACP syndrome and acquired OA, PRG4 dysregulation drives disease initiation and progression, validating its potential as a therapeutic target for DMOAD development.

Although recombinant PRG4 supplementation and gene therapy show encouraging preclinical efficacy, clinical translation is hindered by limited intra−articular retention, difficult recombinant production, insufficient model predictivity, and gene therapy safety concerns. Future research should focus on elucidating the precise regulatory networks of PRG4 expression, optimizing delivery systems to enhance joint retention and functional stability, developing glycosylation−optimized recombinant PRG4, and establishing advanced 3D organoid or ex vivo human joint models to better evaluate disease−modifying effects. Further in−depth investigation of PRG4’s multifaceted functions will not only advance our understanding of OA pathogenesis but also accelerate the development of safe, effective, and clinically translatable PRG4−based therapies for osteoarthritis.
