# Correction: Lubricin in osteoarthritis: functions and therapeutic prospects

**DOI:** 10.3389/fimmu.2026.1964226

**Published:** 2026-08-21

**Authors:** Haochen Li, Guoliang Yi, Dan Zhou, Jiayi Zhou, Zhi Cui, Haowei Zhang, Zhiwei Chen

**Affiliations:** 1 Department of Orthopedic Surgery, The First Affiliated Hospital of University of South China, Hengyang, Hunan, China; 2 The Seventh Affiliated Hospital, University of South China (Hunan Provincial Veterans Administration Hospital), Changsha, Hunan, China

**Keywords:** chondrocyte metabolism, clinical transformation, joint lubrication, lubricin, osteoarthritis, synovial fibrosis, synovial immune microenvironment

Equal contribution statement

Author “Yi Guoliang” was erroneously omitted as equal contributing author. The original version of this article has been updated.

